# Liver biopsy in type 2 diabetes mellitus: Steatohepatitis represents the sole feature of liver damage

**DOI:** 10.1371/journal.pone.0178473

**Published:** 2017-06-01

**Authors:** Mario Masarone, Valerio Rosato, Andrea Aglitti, Tommaso Bucci, Rosa Caruso, Teresa Salvatore, Ferdinando Carlo Sasso, Marie Francoise Tripodi, Marcello Persico

**Affiliations:** 1 Internal Medicine and Hepatology Unit, University of Salerno, Salerno, Italy; 2 Internal Medicine and Hepatology Department, University of Campania “L. Vanvitelli”, Naples, Italy; University of Basque Country, SPAIN

## Abstract

Recent studies report a prevalence of non-alcoholic fatty liver disease (NAFLD) of between 70% and 80% in patients with metabolic syndrome (MS) and type 2 diabetes mellitus (T2DM). Nevertheless, it is not possible to differentiate between simple steatosis and non-alcoholic steatohepatitis (NASH) with non-invasive tests. The aim of this study was to differentiate between simple steatosis and NASH by liver biopsy in patients with hypertransaminasemia and MS or T2DM. Two hundred and fifteen patients with increased ALT levels and MS, and 136 patients at their first diagnosis of T2DM regardless of ALT values were consecutively admitted to a tertiary hepatology center between January 2004 and November 2014. Exclusion criteria were other causes of liver disease/ALT increase. Each patient underwent a clinical, laboratory and ultrasound evaluation, and a liver biopsy. Gender distribution, age, and body mass index were similar in the two groups of patients, whereas cholesterol levels, glycemia and blood pressure were significantly different between the two groups. The prevalence of NAFLD was 94.82% in MS patients and 100% in T2DM patients. NASH was present in 58.52% of MS patients and 96.82% of T2DM. Consequently, this study reveals that, by using liver biopsy, almost all patients with T2DM or MS have NAFLD, which in patients with T2DM means NASH. Importantly, it suggests that NASH may be one of the early complications of T2DM due to its pathophysiological correlation with insulin resistance.

## Introduction

Liver diseases encompass a wide range of clinical signs and histological damage that lead to different degrees of necrosis, inflammation and fibrosis, of which the pathological accumulation of fat in the liver cell (namely steatosis) is often considered one of the first steps of an evolving chronic process [1,2]. Liver steatosis is frequently reported in patients affect by obesity or type 2 diabetes mellitus (T2DM) [3]. The main pathophysiological mechanisms underlying liver steatosis are, at intracellular level, mitochondrial alterations that are also involved in insulin resistance (IR) [4]. These alterations represent the pathophysiological link to the clinical manifestations (hypertension and dyslipidemia) of metabolic syndrome (MS) [5]. The latter syndrome is a cardiovascular disease risk factor. Liver steatosis has been indicated as a cardiovascular risk factor even irrespective of the presence of MS [6] and is significantly related to biological parameters of IR [7].

A diagnosis of liver steatosis (without a history of alcohol abuse, toxic exposure, medication or metabolic disorders), also known as “non-alcoholic fatty liver disease” (NAFLD), is made in case of at least two clinical and histological entities: simple fat accumulation in the liver (“simple steatosis” or “non-alcoholic fatty liver” NAFL) and non-alcoholic steatohepatitis (NASH), which is characterized by fat accumulation, necroinflammation, cellular ballooning, and different stages of liver fibrosis, up to cirrhosis [2]. Simple steatosis and NASH differ in terms of their evolution over time, simple steatosis being a condition that generally does not progress to advanced fibrosis, whereas NASH can progress to liver cirrhosis and/or hepatocellular carcinoma (HCC) [2]. Thus, the differential diagnosis between these two entities is necessary in order to characterize and appropriately monitor patients who are at risk [8].

A series of studies, most of which were based on the non-invasive diagnosis of hepatic steatosis, reported that up to 70–80% of patients with MS and/or T2DM have NAFLD-like features [9–12]. Although clinical and ultrasound signs can identify liver cirrhosis, it is not possible to stage liver steatosis/steatohepatitis using the currently available non-invasive tests [13], and thus patients should undergo a histological examination for staging purposes [14]. Reliable staging of NAFLD is crucial because T2DM is an independent risk factor for HCC in NAFLD, also in non-cirrhotic patients [15–17]. Finally, opinions differ as to whether or not NAFL could chronically evolve and necessarily lead to steatohepatitis and cirrhosis [18]. Therefore, it is conceivable that steatosis might be considered the first insult to the liver that might evolve to liver fibrosis/cirrhosis depending on the patient’s genetic background, their dietetic habits and/or the underlying main disease (i.e., T2DM or MS) [18].

The aim of this study was to assess and stage by liver biopsy steatosis/NASH in patients with hypertransaminasemia and a diagnosis of MS or T2DM.

## Materials and methods

### Patients

**Fig 1:**
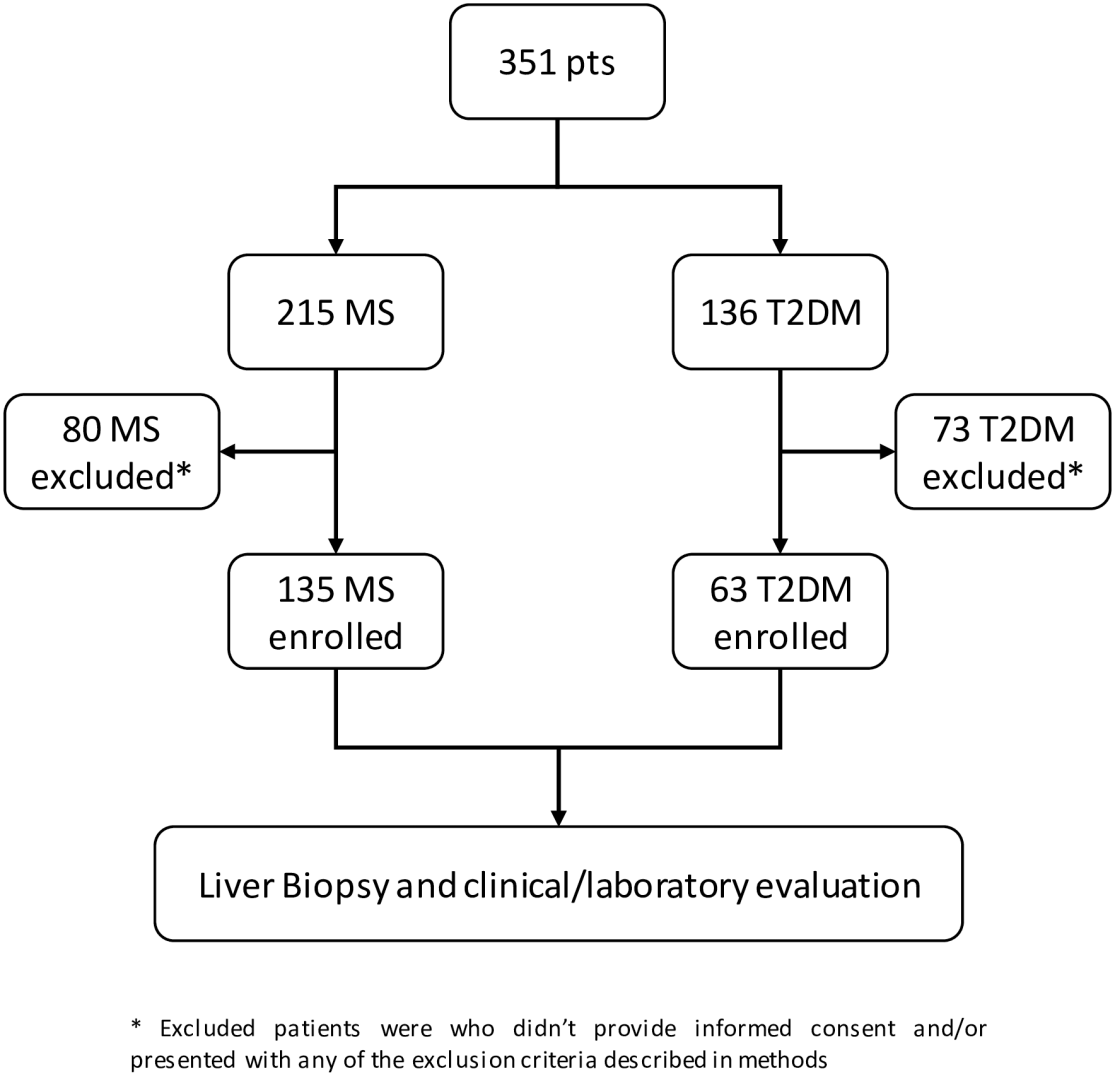
Flow chart of the present study protocol.

As shown in Fig 1, 351 patients (215 with MS and 136 with T2DM) consecutively admitted to a tertiary center of Internal Medicine and Hepatology were evaluated for enrollment in this study between 1 January 2004 and November 2014. Inclusion criteria were: age >18 years, a clinical history (at least 3 evaluations taken within 1 month of each other) of ALT above normal values (>35 U/mL in males and >15 U/mL in females) and a diagnosis of MS according to ATPIII criteria [19] or the first diagnosis of T2DM according to ADA criteria [20]. Exclusion criteria were any known cause of ALT increase, viral hepatitis (HCV, HBV, HSV, EBV or CMV), autoimmune disease, primary or secondary hemochromatosis, Wilson disease, Budd-Chiari disease, cardiac cirrhosis, elevated alcohol intake (>20 grams in females and >30 grams in males per day), and/or celiac disease. Active intravenous drug abuse and/or a history of pharmacotherapy with drugs capable of inducing ALT derangement in the three months prior to admission.

All patients without clinical/laboratory/ultrasound signs of liver cirrhosis (i.e. low platelet count, esophageal varices, spleen enlargement, caudate lobe hypertrophy at ultrasound examination) were asked to undergo liver biopsy. A total of 135/215 MS patients and 63/136 T2DM patients provided written informed consent to liver biopsy and enrolled in the present study. All enrolled patients underwent clinical, laboratory and ultrasound examinations. This study was approved by the Ethics Committee of the University of Campania “L. Vanvitelli”, and was conducted in agreement with the Helsinki Declaration 1975.

### Laboratory and virology tests

Upon hospital admission, each patient underwent a physical examination (body mass index [BMI], blood pressure and waist circumference [WC] measurements), and ultrasound examination of the abdomen, and blood samples were collected for fasting glucose and insulin, HbA1C, triglycerides, HDL cholesterol measurement, complete blood count, ALT and AST, routine biochemistry assay, hepatitis B surface antigen (HBsAg), hepatitis B core antibody (HBcAb), hepatitis A virus antibody (HAVAb) IgM (antibodies by ELISA, Orthodiagnostic system), hepatitis C antibody (HCVAb) (with commercial enzyme linked immunosorbent assay III, Abbot Laboratories Chicago), herpes simplex virus (HSV), anti-nuclear autoantibodies (ANA), anti-mitochondrial antibodies (AMA), smooth muscle antibodies (SMA), and liver kidney microsomal antibodies (LKM). In patients without a diagnosis of T2DM, we calculated the HOMA-IR index and assessed MS according to ATPIII criteria [19,21].

### Liver biopsy and histological examination

Ultrasound guided biopsy was performed in the 135 MS and 63 T2DM patients who provided informed consent, and specimens were obtained from the right hepatic lobe using 17-gauge Menghini modified needles inserted through the intercostal space. The biopsy specimens were formalin-fixed, paraffin-embedded and stained with hematoxylin-eosin, Red Sirius, Ubiquitin, trichrome and Prussian blue. All liver biopsies were read by two experienced pathologists (TS and MP), with an inter-operator diagnostic concordance k >0.8. Liver samples measuring ≥15 mm in length were deemed suitable for histologic analysis when they contained at least 5 entire portal spaces; samples <15 mm were not analyzed. Biopsies were evaluated with the Kleiner score [14] for necroinflammation grading, fibrosis staging and NASH activity score (NAS), and with the Brunt score [22] for the presence and extent of steatosis. Steatosis was graded 1 (<33% of hepatocytes), 2 (33–66% of hepatocytes) or 3 (>66% of hepatocytes), based on the number of fat-replete hepatic cells per microscopic field. Lipid vacuoles were divided according to size, and designated “micro-vacuolar”, “macro-vacuolar” or “mixed”. The minimal histological criteria for a diagnosis of NASH were: macrovescicular steatosis, hepatocyte ballooning, Mallory hyaline bodies, mixed lobular inflammation with polymorphonuclear leucocytes, zone 3 acinar pericellular fibrosis and/or perivenular fibrosis [1,23].

### Statistical analysis

Statistical analyses were performed using the Statistical Program for Social Sciences (SPSS^®^) ver.16.0 for Macintosh^®^ (SPSS Inc., Chicago, Illinois, USA). The Student t-test and the Mann-Whitney U test were performed to compare continuous variables, chi-square with Yates correction or Fisher-exact test to compare categorical variables. Data were reported as mean ± standard deviation for continuous variables with a normal distribution and as median and interval for those with “non-normal” distribution. Statistical significance was defined when p<0.05 in a two-tailed test with a 95% confidence interval.

## Results

The demographic, clinical and laboratory findings of our study population are reported in Table 1. The two groups of patients were comparable in terms of gender distribution, age and BMI. The levels of ALT and parameters related to IR (hypertension, total and HDL cholesterol) were significantly higher in MS patients. The significantly higher ALT levels (p = 0.0014) in MS patients is due to the large number of T2DM patients with normal ALT levels. The histological diagnosis in the two groups of patients is reported in Table 2. The prevalence of NASH and cirrhosis was higher in patients with T2DM than in patients with MS (p<0.0001). NASH was diagnosed in 61/63 (98.6%) of patients with T2DM and in 58.52% patients with MS.

**Table 1:** Demographical, clinical and laboratory parameters of study population. (Data presented as mean (+ standard deviation) except where indicated differently.* Cirrhosis diagnosis: 3 clinical, 13 by liver biopsy.

	MS	T2DM	p
**Screened patients** (n)	215	136	-
**Liver Biopsy** (n)	135	63	**0.002**
**M/F** (n)	80/55	33/30	0.036
**AGE** (years)	54.85 ±14.33	57.27 (±10.50)	0.955
**BMI** (kg/sqm)	33.8 (±6.8)	34.4 (±6.3)	0.473/
**Waist Circumference** (cm)	92.4 (±7.6)	93.2 (±9.3)	0.272
**CHOL** (mg/dL)	189.06 (±50.73)	182.27 (±31.85)	0.163
**HDL** (mg/dL)	37.79 (±13.59)	41.61(±10.46)	**0.005**
**TRI** Median (interval) (mg/dL)	151 (104–208)	159 (115–238)	0.178
**HYPERTENSION** (Y/N)	71/64	20/43	**0.01**
**Glucose** (mg/dL)	90.92 (±23.07)	148.07 (±50.51)	**<0.0001**
**HOMA** Median (interval)	4.1 (2.5–5.8)	-	**-**
**ALT** (U/L)	143.41(±155.36)	45.33 (±29.99)	**<0.0001**
**AST** (U/L)	140.86 (±131.28)	41.27 (±29.23)	**<0.0001**
**GGT** (U/L)	85.71 (±53.94)	81.34 (±50.86)	0.459
**Total bilirubin** (U/L)	1.22 (±0.45)	1.18 (±0.47)	0.426
**HBV** (n)	0	0	-
**HCV** (n)	0	0	-
**Cirrhosis** (n)	0	16 (3+13)*	-

**Table 2:** Prevalence of NAFLD, NASH and cirrhosis in patients undergone to liver biopsy. (NA: Not Applicable).

	MS	DM	OR (95%CI)	p
**N**	135	63	-	-
**NAFLD n (%)**	128/135 (94.82%)	63/63 (100%)	0.001 (0.001–1.138)	0.1
**NASH**	79/135 (58.52%)	61/63 (96.82%)	21.620 (5.58–83.11)	**<0.0001**
**Cirrhosis**	0/135	13/63	NA	**<0.0001**

**Fig 2:**
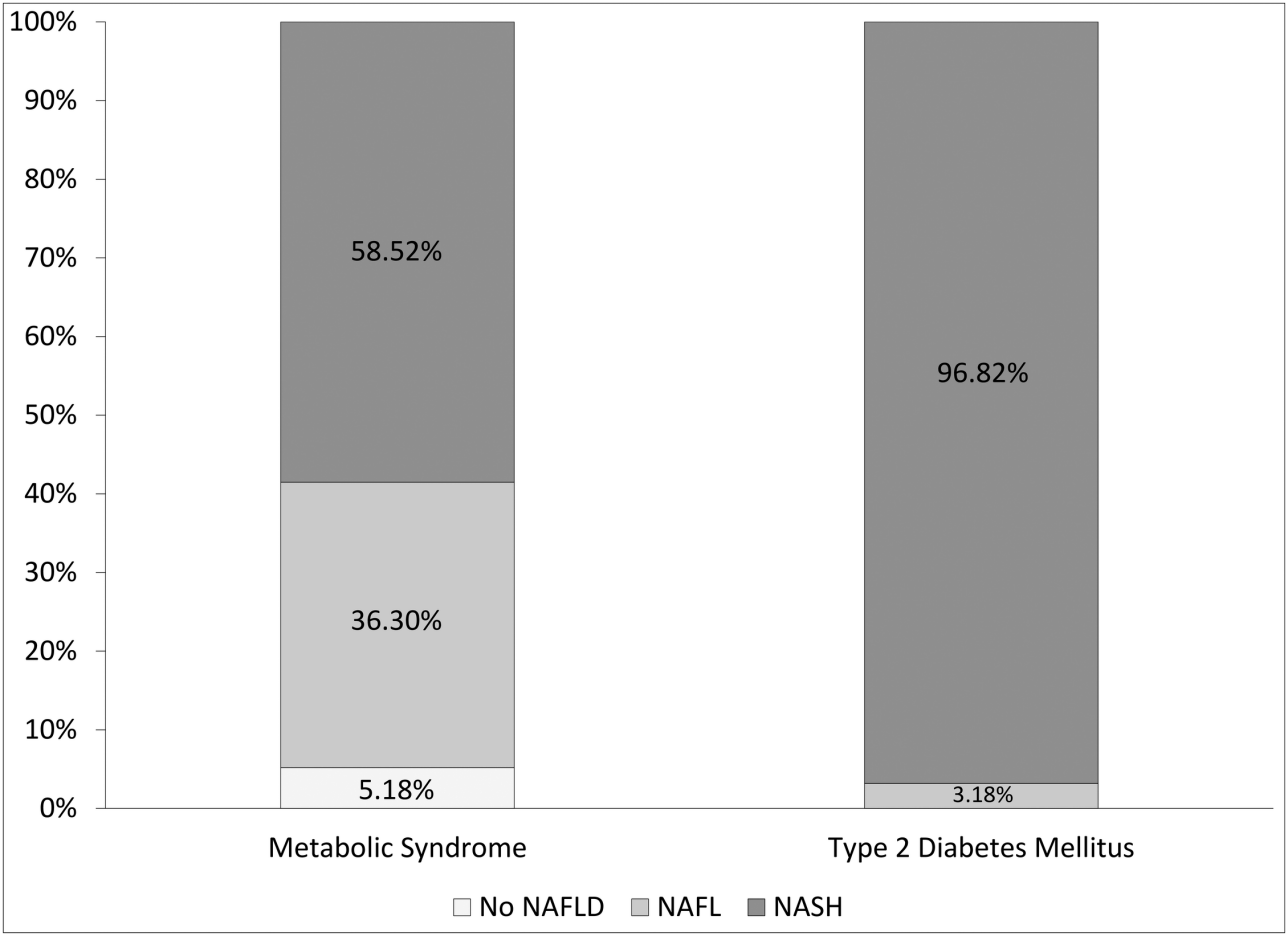
Distribution of the histological diagnosis on the basis of liver biopsy (NAFL, non-alcoholic fatty liver: Simple steatosis without necroinflammation and fibrosis; NASH: Non-alcoholic steatohepatitis; No NAFLD: No histologic signs of steatosis/steatohepatitis).

Prevalence of NAFLD and NASH reported in Fig 2 is in line with the data reported in Table 2. In particular, All T2DM patients had NAFLD, and 96.82% had NASH. Moreover, 7/135 (5.18%) MS patients had neither simple steatosis nor steatohepatitis signs at liver biopsy. Five of these 7 patients had a normal liver biopsy, whereas 2 had a histological presentation in which the only notable alteration was mild enlargement of hepatic sinusoids, which is compatible with a very mild presentation of “peliosis hepatis” [24], a rare type of liver injury that has been associated with a variety of causes, from drugs and chemotherapeutics, infections (including AIDS), neoplasms, malnutrition, cadmium intoxication, anabolic steroids use, myelo-lymphoproliferative disorders, and liver and renal transplantation [25]. Of note, neither of these two patients had any of these previously reported causes. The two “peliosis” patients and subjects with a normal liver biopsy were investigated for any possible extrahepatic cause of ALT derangement and none was found (i.e., no autoimmune/traumatic muscular damage, no cardiac diseases, no drug use, misuse or intoxication, no thyroid dysfunctions etc.).

**Fig 3:**
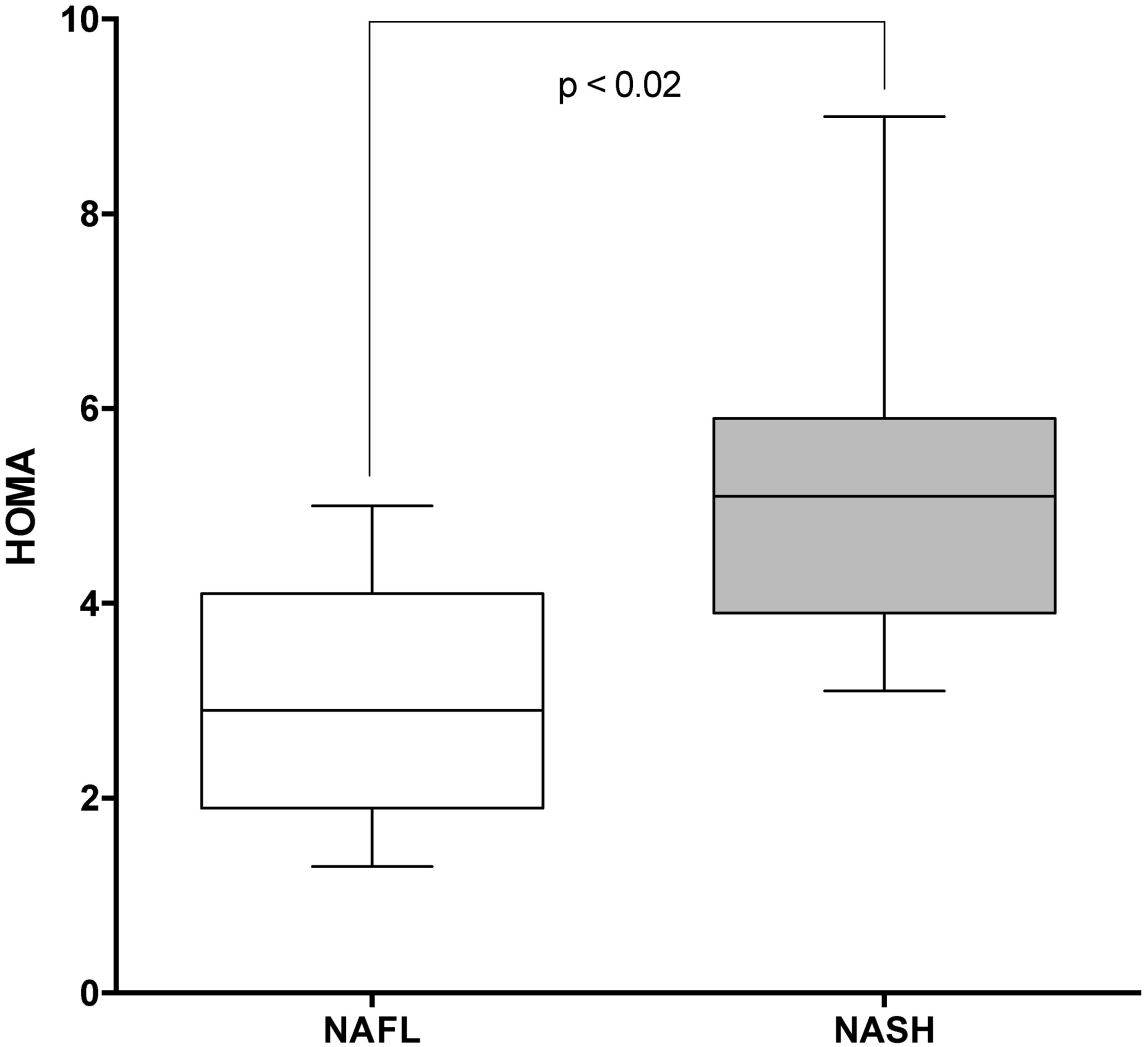
HOMA-IR score in NAFL and NASH patients. *The HOMA-IR score in both NAFL and NASH patients exceeded the diagnostic criteria for insulin resistance.

The HOMA scores of patients with simple steatosis and NASH are reported in Fig 3. HOMA scores were significantly higher in NASH patients than in patients with simple steatosis (p<0.01). Notably, the HOMA scores of these two groups were above the value considered to be diagnostic for IR in Italy [21]. Lastly, as reported in Table 3, the histological activity scores evaluated according to Kleiner et al, [14] for NAFL/NASH were significantly higher in MS patients than in T2DM patients.

**Table 3:** Histological scores [mean±SD] according to Kleiner score in our study population. (*NAS: NAFLD activity score).

	MS	DM	p
**Steatosis [0–3]**	1.6 ± 0.7	2,5 ± 1.3	**<0.0001**
**Lobular Inflammation [0–3]**	0.9 ± 0.7	2.3 ± 1.1	**<0.0001**
**Hepatocellular Ballooning [0–2]**	1.1 ± 0.2	1.2 ± 1.2	ns
**Fibrosis [0–4]**	2.0 ± 0.8	3.1 ± 1.1	**<0.0001**
NAS*	5.6 ± 2.4	10.0 ± 4.7	**<0.0001**

## Discussion

Non-alcoholic fatty liver disease is considered a benign disease, mostly related to the different clinical expression of IR, which ranges from altered lipid metabolism to MS and/or T2DM [8]. While simple steatosis can safely be considered a non-evolutive condition, steatohepatitis (NASH) should not be considered a benign disease because it may evolve into liver cirrhosis and/or HCC [2, 16, 19]. The presence of liver steatosis is easily diagnosed by ultrasound examination, with high sensitivity and specificity [8,9,26]. However, echography is not a reliable method with which to stage the evolution and the degree of liver fibrosis in steatohepatitis, and liver biopsy remains the gold standard for this purpose [27]. Moreover, ultrasonography often fails to identify steatosis that affects less than 20% of hepatocytes [27]. Consequently, the prevalence of NAFLD in MS and T2DM patients, although very high (above 70% to 80%), was often identified with indirect diagnostic methods that may have underestimated the burden of the problem [12]. Moreover, although liver biopsy is considered an invasive practice, Gaidos et al demonstrated that a histological evaluation of NAFLD patients resulted in a survival benefit. In fact, by applying a statistical modeling system liver biopsy outperformed a no-biopsy approach with respect to both mortality and progression to severe disease in NAFLD [28].

It is important to determine whether MS and T2DM patients are affected by simple steatosis or by steatohepatitis in order to assess their prognosis, both in terms of risk of developing liver cirrhosis and in terms of cardiovascular risk [29–31]. In this context, we performed the present study to diagnose and stage liver steatosis in a cohort of patients with MS and increased ALT levels or T2DM upon diagnosis. A large number of patients consented to liver biopsy, which enabled us to directly evaluate the liver disease at histological level. There is a close link between liver steatosis and IR-related metabolic derangements and their complications in both MS and T2DM patients [11,29,30]. The question arises as to whether or not it is possible to predict a progressive chronic pathological mechanism in which, the clinical severity of IR, parallels the clinical severity of liver steatosis.[30] The results reported herein support this concept of liver damage associated to a progressively more severe clinical manifestation of IR. Indeed, we found significantly higher HOMA scores in NASH patients than in patients with simple steatosis (Fig 3). Moreover, the prevalence of NAFLD increased as HOMA levels increased and eventually affected 100% of all T2DM patients, 98% of whom were affected by NASH at histological level. Moreover, 12% of T2DM patients had liver cirrhosis at the histological evaluation, which confirms that the clinical severity IR parallels the degree of liver damage.

We are aware that the prevalence of NAFLD and NASH was very high in our MS and T2DM patients. The 60% prevalence of NASH in MS patients may reflect the fact that 94.81% of these patients had a NAFLD. The prevalence of NASH in NAFLD is reported to be about 60% [12]. The vast majority of studies that have investigated this high prevalence, irrespective of the diagnostic method used, assessed the prevalence of the metabolic syndrome and/or diabetes in NAFLD and not the opposite [12]. Notably, NAFLD itself is generally considered a risk factor for MS and also a consequence of it [29]. In this context, we show that even if not all NAFLD patients may have MS, almost all MS patients may have NAFLD. Regarding T2DM, it has also been frequently reported that NAFLD predicts T2DM and that the studies based on liver biopsy reported higher prevalence rates of NAFLD versus laboratory-based or imaging-based studies [12,32,33] Finally, as mentioned above, our data may be less surprising considering that deranged ALT levels are a not reliable method with which to discriminate between NASH and NAFLD, particularly in diabetic patients[13].

The limitations of this study are its observational and cross-sectional nature. Therefore, we cannot give any insight on the natural history of our patients. Nevertheless, it is noteworthy that almost all the T2DM patients were affected by NASH.

In conclusion, steatohepatitis might be considered the onset of the liver damage in T2DM patients. Thus, it is conceivable that steatosis, in T2DM, is part of a simultaneous process encompassing fat accumulation, chronic inflammation and liver fibrosis that results in liver damage. Given the mounting evidence that NAFLD affects the risk of developing T2DM [29], we can consider that NAFLD, and in particular NASH, are specific clinical signs of T2DM. Routine follow-up with ultrasound examination, and recommending liver biopsy in diabetic patients at the first diagnosis, may be useful tools with which to assess the onset of the disease, to predict overall survival and to evaluate the risk of developing liver cirrhosis, HCC and cardiovascular disease.

## Abbreviations

HCC: hepatocellular carcinoma
HOMA: homeostasis model assessment
IR: insulin resistance
NAFL: non-alcoholic fatty liver
NAFLD: non-alcoholic fatty liver disease
NASH: non-alcoholic steatohepatitis
